# Budesonide/Formoterol Enhances the Expression of Pro Surfactant Protein-B in Lungs of COPD Patients

**DOI:** 10.1371/journal.pone.0083881

**Published:** 2013-12-26

**Authors:** Soo Jung Um, Stephen Lam, Harvey Coxson, Shu Fan Paul Man, Don D. Sin

**Affiliations:** 1 UBC James Hogg Research Centre for Cardiovascular and Pulmonary Research, St. Paul’s Hospital/Providence Health Care, University of British Columbia, Vancouver, BC, Canada; 2 Department of Medicine (Division of Respiratory Medicine), University of British Columbia, Vancouver, BC, Canada; 3 BC Cancer Agency, University of British Columbia, Vancouver, BC, Canada; 4 Department of Radiology, University of British Columbia, Vancouver, BC, Canada; 5 Department of Medicine (Division of Respiratory Medicine), Dong-A University, Busan, Korea; University of Toronto, Canada

## Abstract

**Rationale & Aim:**

Pulmonary surfactants are essential components of lung homeostasis. In chronic obstructive pulmonary disease (COPD), surfactant expression decreases in lungs whereas, there is a paradoxical increase in protein expression in plasma. The latter has been associated with poor health outcomes in COPD. The purpose of this study was to determine the relationship of surfactants and other pneumoproteins in bronchoalveolar lavage (BAL) fluid and plasma to airflow limitation and the effects of budesonide/formoterol on this relationship.

**Methods:**

We recruited (clinical trials.gov identifier: NCT00569712) 7 smokers without COPD and 30 ex and current smokers with COPD who were free of exacerbations for at least 4 weeks. All subjects were treated with budesonide/formoterol 400/12 µg twice a day for 4 weeks. BAL fluid and plasma samples were obtained at baseline and the end of the 4 weeks. We measured lung-predominant pneumoproteins: pro-Surfactant Protein-B (pro-SFTPB), Surfactant Protein-D (SP-D), Club Cell Secretory Protein-16 (CCSP-16) and Pulmonary and Activation-Regulated Chemokine (PARC/CCL-18) in BAL fluid and plasma.

**Results:**

BAL Pro-SFTPB concentrations had the strongest relationship with airflow limitation as measured by FEV_1_/FVC (Spearman rho = 0.509; p = 0.001) and FEV_1_% of predicted (Spearman rho =  0.362; p = 0.028). Plasma CCSP-16 concentrations were also significantly related to airflow limitation (Spearman rho = 0.362; p = 0.028 for FEV_1_% of predicted). The other biomarkers in BAL fluid or plasma were not significantly associated with airflow limitation. In COPD subjects, budesonide/formoterol significantly increased the BAL concentrations of pro-SFTPB by a median of 62.46 ng/ml (p = 0.022) or 48.7% from baseline median value.

**Conclusion:**

Increased severity of COPD is associated with reduced Pro-SFTPB levels in BAL fluid. Short-term treatment with budesonide/formoterol increases these levels in BAL fluid. Long term studies will be needed to determine the clinical relevance of this observation.

## Introduction

The human and economic burden of Chronic obstructive pulmonary disease (COPD) are rapidly growing worldwide [1]. Despite the improved understanding of its pathophysiology and management, it is the only major cause of mortality for which the death rate continues to climb [2], [3]. The advent of novel therapies has been impeded to a certain extent by the paucity of surrogate markers that can be used in early clinical studies and trials to evaluate the therapeutic potential of promising compounds. To overcome this limitation, there has been a great deal of interest in the identification and validation of biomarkers for use in COPD [4], [5]. However, to date there are no well accepted COPD specific biomarkers that have fully addressed this limitation. One potential solution is to focus on lung-specific proteins (which are sometimes referred to as pneumoproteins). Because it is highly unlikely that one molecule can be a biomarker for all relevant domains of COPD (e.g., disease severity, disease activity and therapeutic responsiveness), an alternative approach is to interrogate the performance characteristics of these pneumoproteins for each of these domains, separately. Finally, to date, most of the biomarker studies in COPD have focused on non-invasive sources (e.g. blood or sputum). Very few studies have interrogated bronchoscopic specimens. Thus, it is not certain which of the pneumoproteins, if any, may have biomarker potential in bronchoalveolar fluid, which can be obtained through bronchoscopy. The primary purpose of this study was to determine the relationship of certain pneumoproteins in BAL fluid and plasma to airflow limitation and the impact of inhaled budesonide and formoterol combination on the BAL and plasma concentrations of these pneumoproteins to determine which if any of these biomarkers are responsive to anti-inflammatory drugs commonly used in COPD.

## Materials and Methods

### Study Cohort

We recruited 40 participants in this study. Thirty two subjects had a clinical diagnosis of COPD defined on the basis of forced expiratory volume in one second (FEV_1_) to forced vital capacity (FVC) ratio of less than 70% and FEV_1_ that was less than 80% of predicted following bronchodilation. Eight subjects without COPD (FEV_1_/FVC ratio ≥70%) were also recruited as controls. Inclusion criteria were ≥40 years of age, heavy smokers defined by more than 30 pack-years of smoking, and were free of exacerbation (for those with COPD) and free of any respiratory tract infection (for non-COPD subjects) for more than 4 weeks prior to enrollment. Subjects who were using any inhaled or systemic corticosteroids within 6 months of recruitment or who had a significant medical condition that precluded follow-up such as acute or chronic respiratory failure, cardiovascular instability, and bleeding disorder were excluded. Following informed consent, all subjects underwent spirometry, venipuncture and bronchoscopy at baseline and then at 4 weeks later. During these 4 weeks, they were treated with inhaled budesonide/formoterol (Symbicort Turbuhaler®) 400/12 µg twice daily. Spirometry was performed according to ATS/ERS recommendations [6]. This study was conducted according to the Declaration of Helsinki and Good Clinical Practice guidelines. All enrolled subjects provided written informed consent and the study was approved by the University of British Columbia/Providence Health Care Research Ethics Board (approval number H11-00313).

### Sampling and Measurements

Bronchoscopy with bronchoalveolar lavage (BAL) fluid and blood samples were obtained at baseline and then repeated at the end of the 4 weeks from the same segment. The full details of the procedure have been published previously [7]. Briefly, after canalization, 20 ml of normal saline is instilled into a segmental bronchus in the upper lobe. The fluid is then aspirated back and discarded in order to minimize contamination of the distal BAL by oral secretions. Next, 40 ml of normal saline is instilled, followed by additional instillations of 20 ml aliquots until 30 ml of BAL fluid is recovered, which represents 30% to 50% of the total instilled fluid volume. The The recovered BAL fluid from the distal airspaces was filtered through one layer of sterile gauze swab and centrifuged at 242 g for 10 minutes at 4°C. The supernatant was decanted and divided into aliquots for storage at −80°C until measurements. Blood sample was taken from a peripheral vein and collected in 10 mL glass tubes containing ethylenediaminetetraacetic acid. Plasma was then separated and stored at −80°C. The BAL fluid and plasma samples were thawed once. From these samples (both baseline and 4 week samples), we measured lung predominant proteins, pro-SFTPB, SP-D, CCSP-16, and PARC/CCL-18 The study personnel who performed the measurements were blinded to the date of sample procurement and the baseline and 4 week samples (from the same patients) were aliquoted into the same plates to abrogate “batch effect”. Pro-SFTPB (synthesized in-house by generating anti-pro-SFTPB mouse monoclonal antibodies against the N-terminal pro-peptide of human SFTPB), SP-D (Bio Vendor Laboratory Medicine, Modrice, Czech Republic), CCSP-16 (Bio Vendor Laboratory Medicine, Modrice, Czech Republic), PARC/CCL-18 (R&D Minneapolis, MN, USA) were determined using commercially available ELISA kits according to the manufacturer’s instructions. All of these analytes were measured in duplicate with a coefficient of variation of 2.2% for pro-SFTPB, 4.4% for SP-D, 1.9% for CCSP-16, and 3.9% for PARC/CCL-18 from BAL samples, 6.7%, 5.9%, 2.8%, and 1.5% from plasma samples respectively. The analytical limit of detection for the pro-SFTPB was 2.74 pg/ml, that for SPD was 0.01 ng/mL, that for CCSP-16 was 0.65 ng/mL, and that for PARC/CCL-18 was 10 pg/mL. The specificity of pro-SFTPB was confirmed using mass spectrometry and western blotting (data not shown).

### Statistical methods

Continuous variables with normal and skewed distribution are presented as mean ± standard deviation or median and interquartile range, respectively. Categorical variables are presented as frequencies and group percentage. The student t-test and paired sample t-test and the Fisher’s exact test were used for comparison of normally distributed continuous and categorical variables, respectively. The Kolmogorov-Smirnov test was used to test normal distribution of continuous variables. If the normality assumption failed, a Mann-Whitney U test was used for comparison of these continuous variables. The correlation analysis was performed to determine the relationship of BAL and plasma biomarkers to airflow limitation as reflected by FEV_1_% of predicted and FEV_1_/ FVC ratio. We then constructed a multiple regression model to adjust for the possible confounding effects of age, gender, body mass index and pack-years of smoking. All tests were two-tailed and a p-value <0.05 was considered statistically significant. All statistical analysis was performed using SPSS 18 (SPSS Inc., Chicago, IL, USA) software.

## Results

### Study Subjects

We excluded 3 subjects in whom adequate BAL and plasma samples could not be collected. Thus, the final analysis was conducted on 37 patients. The baseline demographic and clinical characteristics are presented in Table 1. The mean age was 64.97±6.29 years, and 21 (56.8%) patients were men. All subjects were heavy smokers with a mean history of smoking was 47.83±16.80 pack-years. Three (8.1%) were current smokers; the rest were ex-smokers. The mean FEV_1_ and FEV_1_/FVC ratio of the study cohort was 73.14±18.30 predicted and 66.30±9.43, respectively. The median concentrations of pro-SFTPB and CCSP-16 in BAL fluid were lower in subjects with COPD compared to those without COPD (271.86 ng/ml vs. 921.55 ng/ml, p = 0.059; 27.62 ng/ml vs. 75.87 ng/ml, p = 0.040).

**Table 1:** Baseline Characteristics of Study Participants.

Groups	Overall	COPD	Control	P value†
No. of subjects	37	30	7	
Age (years)	64.92±6.29	65.10±6.81	64.14±3.48	0.947
Male/female	21/16	14/16	7/0	0.012
Body mass index (kg/m^2^)	26.98±4.03	26.36±3.67	29.66±4.97	0.063
Ex-smoker/current smoker	34/3	27/3	7/0	1.000
Smoking (pack-years)	47.83±16.80	46.36±15.51	54.14±21.75	0.697
FEV_1_% of predicted	73.14±18.30	67.13±10.38	98.86±23.19	<0.001
FEV_1_/FVC	66.30±9.43	62.73±6.37	81.57±1.57	<0.001

FEV_1_, forced expiratory volume in 1 second. FVC, forced vital capacity.

P value denotes comparison between COPD and control subjects.

### BAL biomarkers (Table 2)

**Table 2:** Biomarker Levels in BAL and Plasma at Baseline and at 4 Weeks of Treatment with Inhaled Budesonide/Formoterol Combination.

Groups	Overall	COPD	Control	P value†
No. of subjects	37	30	7	
**Baseline BAL**				
Pro-SFTPB (ng/ml)	286.52(123.65−524.29)	271.86(117.25−396.69)	921.55(242.90−1207.92)	0.059
SP-D (ng/ml)	2.72(1.55−5.71)	2.81(1.71−6.55)	1.86(0.25−3.48)	0.138
CCSP-16 (ng/ml)	30.98(20.59−55.57)	27.62(18.85−44.73)	75.87(23.42−284.01)	0.040
PARC/CCL-18 (pg/ml)	74.55(41.87−196.24)	71.39(41.56−205.48)	78.22(38.93−174.62)	0.894
**Baseline Plasma**				
Pro-SFTPB (ng/ml)	31.90(19.25−56.95)	31.90(16.80−56.93)	29.60(21.70−68.90)	1.000
SP-D (ng/ml)	137.60(74.45−181.00)	139.30(78.20−180.95)	105.70(48.20−229.30)	0.435
CCSP-16 (ng/ml)	5.20(2.85−6.45)	3.45(2.50−5.95)	5.60(5.20−7.00)	0.100
PARC/CCL-18 (pg/ml)	43.90(34.90−52.45)	41.40(31.65−52.85)	45.50(44.70−53.20)	0.149
**4 week BAL**				
Pro-SFTPB (ng/ml)	424.48(208.94−663.71)	405.90(172.91−578.06)	921.55(242.90−1207.92)	0.187
SP-D (ng/ml)	2.68(0.96−4.68)	3.28(1.35−4.86)	1.86(0.25−3.48)	0.175
CCSP-16 (ng/ml)	31.48(16.27−74.44)	27.92(14.42−57.34)	75.87(23.41−284.01)	0.084
PARC/CCL-18 (pg/ml)	111.99(69.50−246.56)	119.96(82.73−249.06)	78.22(38.93−174.62)	0.151
**4 week Plasma**				
Pro-SFTPB (ng/ml)	30.90(22.10−59.90)	29.25(22.55−59.23)	37.80(16.20−82.30)	0.906
SP-D (ng/ml)	114.50(73.70−170.70)	132.45(81.93−172.35)	98.60(59.95−185.05)	0.451
CCSP-16 (ng/ml)	4.30(2.50−6.00)	4.20(2.45−6.03)	5.00(3.70−6.80)	0.311
PARC/CCL-18 (pg/ml)	42.90(30.40−56.80)	41.50(29.80−51.80)	68.10(54.15−86.35)	0.003

BAL, bronchoalveolar lavage. FEV_1_, forced expiratory volume in 1 second. FVC, forced vital capacity. Pro-SFTPB, pro-surfactant protein-B. SP-D, surfactant protein-D. CCSP-16, club cell secretory protein-16. PARC, pulmonary and activation regulated chemokine.

Data are expressed as median and interquartile range.

P denotes the comparison between COPD and Control subjects.

**Figure 1:**
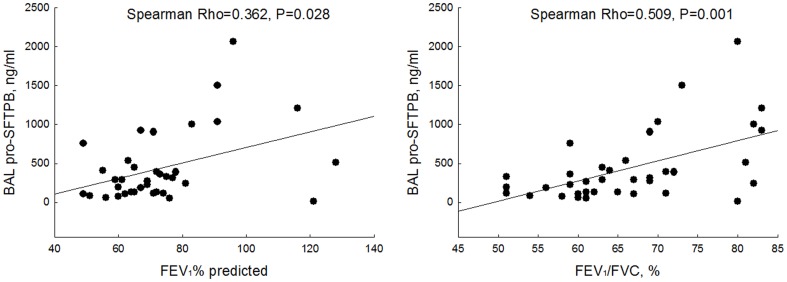
The relationship between baseline BAL pro-SFTPB and lung function. Significant relationship is noted between baseline BAL pro-SFTPB and FEV_1_% predicted, as well as between baseline pro-SFTPB and FEV_1_/FVC ratio.

At baseline, pro-SFTPB concentrations were significantly associated with severity of airflow limitation as measured by FEV_1_ % of predicted (Spearman rho = 0.362, p = 0.028) and FEV_1_/FVC ratio (Spearman rho = 0.509, p = 0.001) (Figure 1). Adjustments for age, sex, cigarette smoking (current versus ex-smokers) and BMI made no material difference to the relationship between pro-SFTPB and FEV_1_/FVC ratio (standardized regression coefficient 0.361; p = 0.028). However, these adjustments made the relationship between pro-SFTPB and FEV_1_ % of predicted non-significant (standardized regression coefficient 0.240; p = 0.185). Other biomarkers in the BAL fluid were not significantly related to airflow limitation. BAL pro-SFTPB showed a significant correlation with only body mass index (Spearman rho = 0.459, p = 0.004) among the clinical variables that were measured (Table 3). BAL pro-SFTPB concentrations were significantly related to other pneumo-biomarkers in the BAL fluid.

**Table 3:** The relationship of the level of BAL pro-surfactant protein-B with clinical characteristics and other BAL pneumoproteins in all subjects.

	Spearman Rho	P value
Age (years)	−0.078	0.648
Female (vs. male)	−0.015	0.928
Smoking (pack-years)	−0.052	0.761
Body mass index (kg/m^2^)	0.459	0.004
FEV_1_% of predicted	0.362	0.028
FEV_1_/FVC	0.509	0.001
SP-D (ng/ml)	0.434	0.007
CCSP-16 (ng/ml)	0.541	0.001
PARC/CCL-18 (pg/ml)	0.566	<0.001

BAL, bronchoalveolar lavage. FEV_1_, forced expiratory volume in 1 second. FVC, forced vital capacity. SP-D, surfactant protein-D. CCSP-16, club cell secretory protein-16. PARC, pulmonary and activation regulated chemokine.

**Figure 2:**
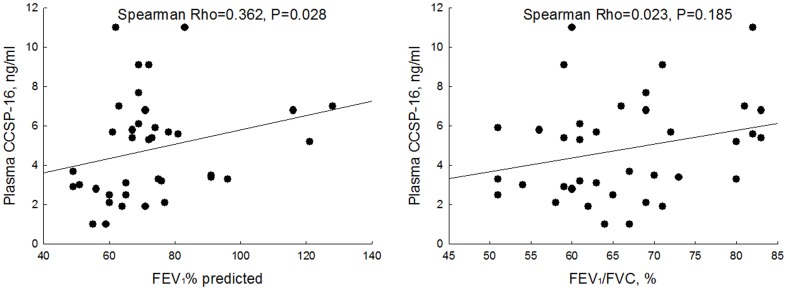
BAL and plasma biomarkers at baseline and 4 weeks after budesonide/formoterol treatment in COPD patients. Budesonide/formoterol significantly increased the BAL concentrations of pro-SFTPB. Data are presented as median with interquartile range and outliers are shown.

In patients with COPD, one month treatment with budesonide/formoterol significantly increased BAL concentrations of pro-SFTPB by a median (interquartile range) of 62.46 ng/ml (−50.62 to 264.04) (p = 0.022), representing a 48.7% from the baseline median value (Figure 2). None of clinical variables affected the change of BAL pro-SFTPB over the treatment period. Other BAL biomarkers did not significantly change over the treatment period (SP-D median −0.20, interquartile range −2.91 to 1.46, p = 0.412; CCSP-16 1.94, −12.70 to 29.69, 0.245; PARC/CCL-18 38.80, −15.43 to 66.20, 0.090).

### Plasma biomarkers (Table 2)

**Figure 3:**
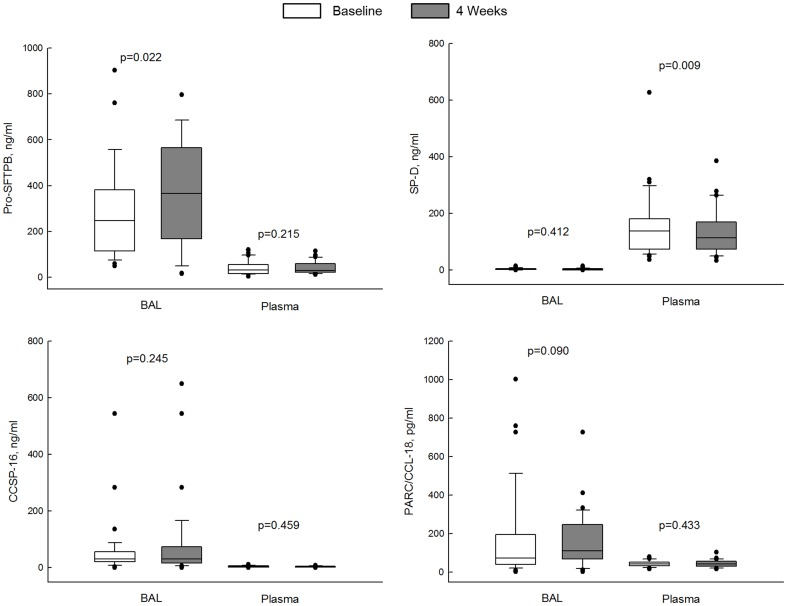
The relationship between baseline plasma CCSP-16 and lung function. Significant relationship is noted between baseline plasma CCSP-16 and FEV_1_% predicted.

Only plasma concentrations of CCSP-16 were significantly associated with lung function (FEV1 % of predicted Spearman rho = 0.362, p = 0.028; FEV_1_/FVC ratio 0.023, 0.185) (Figure 3). The plasma CCSP-16 concentrations were higher in men than women (median 5.70 vs. 3.35, p = 0.047), however, other clinical characteristics and plasma biomarkers did not affect its level.

One month treatment with budesonide/formoterol significantly reduced plasma concentrations of SP-D by a median (interquartile range) of −14.10 ng/ml (−36.68 to 5.10) (p = 0.009). Other plasma biomarkers did not showed a significant difference over the treatment period (change in pro-SFTPB median 1.95, interquartile range −2.88 to 9.78, p = 0.215; CCSP-16 −0.20, −0.90 to 0.60, 0.459; PARC/CCL−18 −2.10, −0.90 to 4.00, 0.433). The changes in the BAL and plasma biomarkers after 1month treatment of budesonide/formoterol are presented in Figure 2.

## Discussion

In this proof of principle study we used both BAL and plasma to interrogate the role (if any) of pneumoproteins as possible biomarkers of lung function and therapeutic responsiveness to an inhaled anti-inflammatory medication. We found that pro-SFTPB concentrations in BAL fluid were significantly related to lung function and that these levels could be increased with the use of inhaled budesonide/formoterol combination. On the other hand, we found that 4 weeks of inhaled budesonide/formoterol significantly reduced plasma concentrations of SP-D, in keeping with our previous observation [8]. Lastly, although plasma CCSP-16 concentrations were significantly related to FEV1%, they did not change significantly with budesonide/formoterol. Together, these data suggest that in plasma, CCSP-16 and SP-D are promising biomarkers for lung function and therapeutic responsiveness to inhaled anti-inflammatory medications, respectively; whereas in the BAL, pro-SFTPB is a promising biomarker for both of these outcomes.

Lung and systemic inflammation are key components of COPD. As the disease progresses, the inflammatory response is amplified [9]. However, because the systemic inflammatory process can be contributed by different organs (and not just the lungs), discovery of COPD “specific” biomarkers has been challenging. To surmount this limitation, recent investigations have focused on proteins that are synthesized mostly in the lung such as surfactant proteins, CCSP-16, and PARC/CCL18.

Surfactants are particularly interesting in that they are important for reducing surface tension at the air–liquid interface of lungs and thus are essential for life [10], [11]. Surfactants are composed mostly of phospholipids, making them largely hydrophobic with few exceptions. The mature (and functional) form of surfactant protein B (SFTPB) is approximately 8 kDa in weight and is extremely hydrophobic [10], [12]. The main function of SFTPB is to accelerate the formation of a surface active film composed of phospholipids at the air-water interface by increasing adsorption rate [12]. SFTPB also has anti-inflammatory properties and may be involved in protecting the lung against oxidative stress [13], [14]. With smoking or acute lung injury, lung BAL protein concentrations of SFTPB decrease [15], [16]. Most importantly, to our knowledge, no extra-pulmonary organs produce any appreciable amount of SFTPB, making SFTPB a highly specific lung biomarker. However, the main limitation of SFTPB as a biomarker is its hydrophobicity, and thus cannot be easily measured in plasma or BAL. Pro-SFTPB, on the other hand, is water soluble, which may explain why it was detectable in the BAL fluid and plasma. SFTPB is synthesized by Type II alveolar cells initially as a hydrophilic 42 kDalton pro-SFTPB and then processed post-translationally into a mature 8 kDa SFTPB through a series of proteolytic cleavages at both the N and C terminus of the protein [17]. The mature form is then secreted into the alveolar space from the lamellar bodies of Type II alveolar cells where they polymerize and associate with surfactant phospholipids, creating the complex surfactant that can reduce surface tension in lungs. Importantly, while SFTPB protein expression can be found in a variety of cells in lungs and elsewhere, SFTPB mRNA expression is localized exclusively to type II alveolar cells and nonciliated epithelial cells. Thus, SFTPB is a highly specific pneumoprotein unlike other surfactants such as SP-D and SP-A, which can be genetically expressed by other cells and organs [18]–[20]. This unique property of SFTPB makes it a very promising biomarker for evaluating disease severity and perhaps even disease activity in COPD and other inflammatory lung diseases. This may explain why despite the relatively small sample size, we found a significant relationship between pro-SFTPB levels in BAL fluid and airflow limitation and a significant increase following 1 month of therapy with budesonide/formoterol combination, which has been shown to improve lung function and health status and to reduce exacerbation risks in patients with COPD.

On the other hand, pro-SFTPB in plasma was not related to airflow limitation and was not responsive to short term therapy. This likely occurred because the plasma expression of pro-SFTB is relatively low (figure 3) even in patients with COPD, reducing its discriminative property as a blood biomarker. In contrast, SP-D levels are higher in plasma than in the BAL fluid, making this a more attractive blood biomarker. Furthermore, plasma SP-D levels are modifiable with short term therapy with combination products [8] and as reported previously, these changes are associated with improved health status in patients with COPD. Thus, SP-D may be a promising blood biomarker to evaluate novel anti-inflammatory drugs in short term COPD studies.

Another promising blood biomarker is CCSP-16, which is secreted predominantly by non-ciliated Clara cells (club cells) in respiratory bronchioles and by non-ciliated columnar epithelial cells of the large and small airways. We found that plasma levels of this protein were associated with lung function, consistent with data reported by others [21]. However, neither BAL fluid nor plasma concentrations of this protein were modifiable with short-term budesonide/formoterol therapy, suggesting that this protein may be a promising biomarker of disease severity but not necessarily of disease activity in patients with COPD. Additional studies will be required to validate this hypothesis.

There were several limitations to this study. First, this was a single centered study with a relatively small sample size. However, the data on SP-D and CCSP-16 from this study were very similar to those of larger multi-centered studies [8], [22]. Second, we did not have a placebo arm to this study. However, the purpose of this study was not to demonstrate the therapeutic efficacy of budesonide/formoterol (which has been shown previously) [23] but to demonstrate the performance characteristics of the selected pneumoproteins in both BAL fluid and plasma. Thus, the presence of a placebo comparator was not critical to this study. Third, all subjects were current or former heavy smokers. Thus these data cannot be generalized to all COPD patients. Fourth, we did not evaluate budesonide and formoterol separately; thus, it is uncertain which components (or both) lead to the changes in plasma concentrations of SP-D or pro-SFTPB changes in BAL. A previous study suggests that circulating SP-D is responsive mostly to inhaled corticosteroids [8].

In conclusion, the findings of the present study suggest that owing to their inherent molecular properties, different pneumoproteins may have different biomarker roles in COPD. In the BAL fluid, pro-SFTPB may provide information on disease severity and responsiveness to anti-inflammatory drugs; whereas in the systemic circulation, CCSP-16 may be a biomarker of disease severity and SP-D may associate with therapeutic responsiveness to inhaled corticosteroids. If these data can be generalized and validated by additional studies, these pneumoproteins may provide new tools for clinicians, researchers and industry to “customize” biomarker panels to evaluate specific endpoints in COPD.

## References

[1] World Health Organization (2008) World Health Statistics. Available: http://www.who.int/gho/publications/world_health_statistics/2008/en/index.html. Accessed 2013 Oct 23.

[2] Lozano R, Naghavi M, Foreman K, Lim S, Shibuya K, et al (2012) Global and regional mortality from 235 causes of death for 20 age groups in 1990 and 2010: a systematic analysis for the Global Burden of Disease Study 2010. Lancet 380: 2095–2128. 23245604 10.1016/S0140-6736(12)61728-0 PMC10790329

[3] Murray CJ, Lopez AD (2013) Measuring the global burden of disease. N Engl J Med 369: 448–457. 23902484 10.1056/NEJMra1201534

[4] Barnes PJ, Chowdhury B, Kharitonov SA, Magnussen H, Page CP, et al (2006) Pulmonary biomarkers in chronic obstructive pulmonary disease. Am J Respir Crit Care Med 174: 6–14. 16556692 10.1164/rccm.200510-1659PP

[5] Tzortzaki EG, Lambiri I, Vlachaki E, Siafakas NM (2007) Biomarkers in COPD. Curr Med Chem 14: 1037–1048. 17439401 10.2174/092986707780362943

[6] Miller MR, Hankinson J, Brusasco V, Burgos F, Casaburi R, et al (2005) Standardisation of spirometry. Eur Respir J 26: 319–338. 16055882 10.1183/09031936.05.00034805

[7] Lam S, LeRiche JC, Kijek K (1985) Effect of filtration and concentration on the composition of bronchoalveolar lavage fluid. Chest 87: 740–742. 3996060 10.1378/chest.87.6.740

[8] Sin DD, Man SF, Marciniuk DD, Ford G, FitzGerald M, et al (2008) The effects of fluticasone with or without salmeterol on systemic biomarkers of inflammation in chronic obstructive pulmonary disease. Am J Respir Crit Care Med 177: 1207–1214. 18310480 10.1164/rccm.200709-1356OC

[9] Hogg JC, Chu F, Utokaparch S, Woods R, Elliott WM, et al (2004) The nature of small-airway obstruction in chronic obstructive pulmonary disease. N Engl J Med 350: 2645–2653. 15215480 10.1056/NEJMoa032158

[10] Pattle RE (1955) Properties, function and origin of the alveolar lining layer. Nature 175: 1125–1126. 14394123 10.1038/1751125b0

[11] Wright JR (2005) Immunoregulatory functions of surfactant proteins. Nat Rev Immunol 5: 58–68. 15630429 10.1038/nri1528

[12] Whitsett JA, Ohning BL, Ross G, Meuth J, Weaver T, et al (1986) Hydrophobic surfactant-associated protein in whole lung surfactant and its importance for biophysical activity in lung surfactant extracts used for replacement therapy. Pediatr Res 20: 460–467. 3754957 10.1203/00006450-198605000-00016

[13] Tokieda K, Ikegami M, Wert SE, Baatz JE, Zou Y, et al (1999) Surfactant protein B corrects oxygen-induced pulmonary dysfunction in heterozygous surfactant protein B-deficient mice. Pediatr Res 46: 708–714. 10590028 10.1203/00006450-199912000-00014

[14] Miles PR, Bowman L, Rao KM, Baatz JE, Huffman L (1999) Pulmonary surfactant inhibits LPS-induced nitric oxide production by alveolar macrophages. Am J Physiol 276: L186–196. 9887071 10.1152/ajplung.1999.276.1.L186

[15] Nguyen AB, Rohatgi A, Garcia CK, Ayers CR, Das SR, et al (2011) Interactions between smoking, pulmonary surfactant protein B, and atherosclerosis in the general population: the Dallas Heart Study. Arterioscler Thromb Vasc Biol 31: 2136–2143. 21817103 10.1161/ATVBAHA.111.228692 PMC3177606

[16] Robin M, Dong P, Hermans C, Bernard A, Bersten AD, et al (2002) Serum levels of CC16, SP-A and SP-B reflect tobacco-smoke exposure in asymptomatic subjects. Eur Respir J 20: 1152–1161. 12449168 10.1183/09031936.02.02042001

[17] Pryhuber GS (1998) Regulation and function of pulmonary surfactant protein B. Mol Genet Metab. 64: 217–228. 10.1006/mgme.1998.2722 9758711

[18] Phelps DS, Floros J (1991) Localization of pulmonary surfactant proteins using immunohistochemistry and tissue in situ hybridization. Exp Lung Res 17: 985–995. 1769356 10.3109/01902149109064330

[19] Murray E, Khamri W, Walker MM, Eggleton P, Moran AP, et al (2002) Expression of surfactant protein D in the human gastric mucosa and during Helicobacter pylori infection. Infect Immun 70: 1481–1487. 11854236 10.1128/IAI.70.3.1481-1487.2002 PMC127735

[20] Rubio S, Lacaze-Masmonteil T, Chailley-Heu B, Kahn A, Bourbon JR, et al (1995) Pulmonary surfactant protein A (SP-A) is expressed by epithelial cells of small and large intestine. J Biol Chem 270: 12162–12169. 7744866 10.1074/jbc.270.20.12162

[21] Vestbo J, Edwards LD, Scanlon PD, Yates JC, Agusti A, et al (2011) Changes in forced expiratory volume in 1 second over time in COPD. N Engl J Med 365: 1184–1192. 21991892 10.1056/NEJMoa1105482

[22] Lomas DA, Silverman EK, Edwards LD, Miller BE, Coxson HO, et al (2008) Evaluation of serum CC-16 as a biomarker for COPD in the ECLIPSE cohort. Thorax 63: 1058–1063. 18757456 10.1136/thx.2008.102574

[23] Welte T, Miravitlles M, Hernandez P, Eriksson G, Peterson S, et al (2009) Efficacy and tolerability of budesonide/formoterol added to tiotropium in patients with chronic obstructive pulmonary disease. Am J Respir Crit Care Med 180: 741–750. 19644045 10.1164/rccm.200904-0492OC

